# Combination of mesenchymal stem cells and cidofovir for the treatment of BK virus-associated late-onset hemorrhagic cystitis after allogeneic hematopoietic stem cell transplantation: a case report

**DOI:** 10.3389/fmed.2026.1865978

**Published:** 2026-09-24

**Authors:** Feifei Liu, Cheng Zhang, Huaping Du

**Affiliations:** Department of Hematology, Sir Run Run Shaw Hospital, School of Medicine, Zhejiang University, Hangzhou, Zhejiang, China

**Keywords:** BK virus, cidofovir, hematopoietic stem cell transplantation, hemorrhagic cystitis, mesenchymal stem cells

## Abstract

This case report describes a 22-year-old female patient who developed refractory grade IV BK virus-associated late-onset hemorrhagic cystitis (HC) on day 69 following haploidentical hematopoietic stem cell transplantation. After conventional stepwise interventions—including bladder irrigation, repeated transcatheter arterial embolization, and endoscopic electrocoagulation—failed to achieve disease control, a combination regimen comprising umbilical cord-derived mesenchymal stem cells (MSCs) and intravenous cidofovir was administered on day 84 post-transplant. Following this combined therapy, the patient's bladder hemorrhage was progressively controlled, dysuria was relieved, and transfusion requirements decreased. Macroscopic hematuria began to decrease on day 98 post-transplant and resolved completely by day 124, accompanied by a marked decline in BK viral load during follow-up. This case suggests that the combination of MSCs and cidofovir may serve as a potential salvage strategy for severe, refractory virus-associated HC and may also offer ancillary benefit in promoting hematopoietic recovery.

## Introduction

Late-onset hemorrhagic cystitis (HC) is a frequent and severe complication after allogeneic hematopoietic stem cell transplantation (allo-HSCT) for hematologic malignancies, primarily driven by BK virus (BKV) reactivation. Grade IV HC, characterized by massive hemorrhage and clot formation, poses a major therapeutic challenge (1). Conventional stepwise management—including immunosuppressant reduction, bladder irrigation, interventional embolization, and endoscopic electrocoagulation—often yields suboptimal results in refractory cases. Although various antiviral agents have been investigated, cidofovir remains one of the better-evidenced options for BKV infection; however, its clinical utility is limited by significant nephrotoxicity and uncertain efficacy as monotherapy (2). Mesenchymal stem cells (MSCs) have recently emerged as a promising therapeutic modality due to the potent immunomodulatory and tissue-repair properties (3, 4). To date, no reports have described the combination of MSCs and cidofovir for severe, refractory HC, partly due to concerns over toxicity and limited drug accessibility. This case report describes the successful management of refractory grade IV BKV-associated HC following haploidentical HSCT using a novel combination of umbilical cord-derived MSCs and intravenous cidofovir after all conventional therapies had failed.

## Case presentation

A 22-year-old woman was diagnosed with early T-cell precursor acute lymphoblastic leukemia (ETP-ALL) in May 2024, confirmed by bone marrow morphology, immunophenotyping, cytogenetics, and molecular biology. She had a history of hypothyroidism managed with levothyroxine 50 μg daily. After two cycles of Hyper-CVAD (regimens A and B) and one additional cycle of Hyper-CVAD A, she achieved minimal residual disease (MRD)-negative complete remission (CR).

On 22 October 2024, she underwent a haploidentical allogeneic hematopoietic stem cell transplantation (father-to-daughter) following a TBF conditioning regimen (thiotepa, busulfan, fludarabine) and post-transplant cyclophosphamide. Graft-versus-host disease (GVHD) prophylaxis consisted of anti-thymocyte globulin, cyclosporine, mycophenolate mofetil, and low-dose methotrexate. Peripheral blood stem cells were infused over 3 days (30 October–1 November), with a total mononuclear cell dose of 15.91 ×  10⁸/kg and a CD34+ cell dose of 6.37 × 10⁶/kg. Neutrophil and platelet engraftment occurred on days 13 and 14, respectively. The early post-transplant course was complicated by bloodstream and gastrointestinal *Candida tropicalis* infection (resolved with liposomal amphotericin B) and drug-induced acute kidney injury (reversed after discontinuation of nephrotoxic agents and continuous renal replacement therapy). On day 15, transient grade II HC resolved with supportive care. Day-30 bone marrow evaluation confirmed MRD-negative CR (Figure 1).

**Figure 1:**
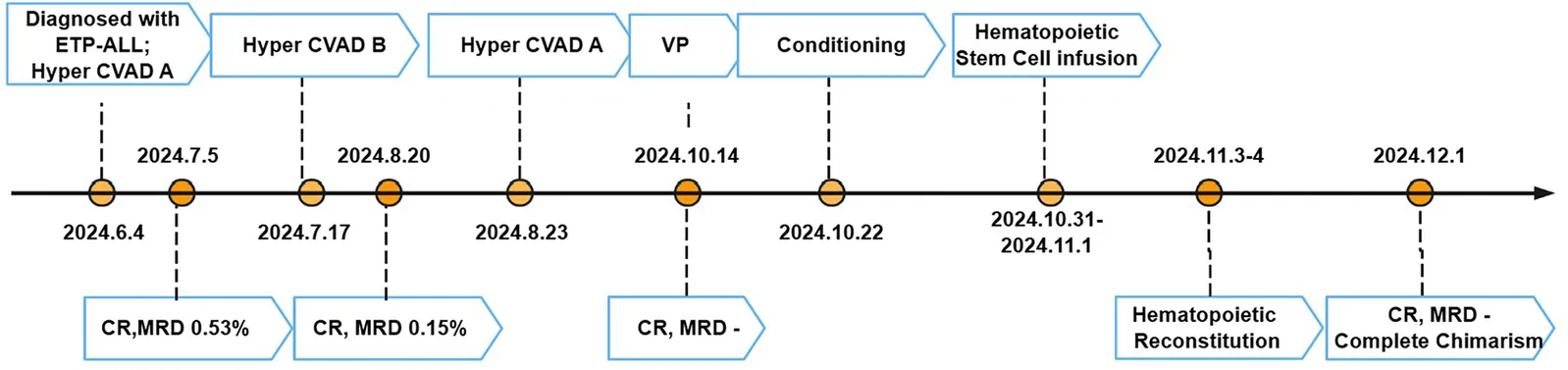
Flowchart of major events before and after transplantation.

From day 30 onward, the patient developed progressive BKV reactivation, with urine BKV load reaching 4.13 ×  10⁹ copies/L. Concurrently, she experienced Epstein–Barr virus reactivation on day 33 (peak 2.52 ×  10⁴ copies/mL), which cleared after two weekly doses of rituximab (days 34 and 41), and cytomegalovirus (CMV) DNAemia on day 47 (7.23 ×  10^3^ copies/mL), which resolved spontaneously.

On day 69, the patient developed grade IV HC (graded according to the NCI CTCAE) with gross hematuria, severe dysuria, and large clot passage. Laboratory findings showed pancytopenia (hemoglobin, 35 g/L; platelets, 40 ×  10⁹/L) and a fibrinogen level of 1.55 g/L. Bladder ultrasound confirmed extensive intravesical clots, and the irrigation fluid (highly diluted urine) tested positive for BKV with a load of 4.22 ×  10⁸ copies/L, establishing the diagnosis of BKV-associated HC. Testing for peripheral blood BKV-DNA was negative. Initial measures-continuous bladder irrigation, aggressive transfusion support (red blood cells, platelets, fresh frozen plasma, cryoprecipitate), and reduction of immunosuppression—failed to control bleeding.

The patient subsequently required three invasive embolization procedures: bilateral vesical artery embolization on day 71, bilateral internal pudendal artery embolization on day 74, and embolization of the bilateral internal iliac artery branches (Figure 2) and cystoscopic electrocoagulation on day 81. Despite these interventions, hematuria persisted.

**Figure 2:**
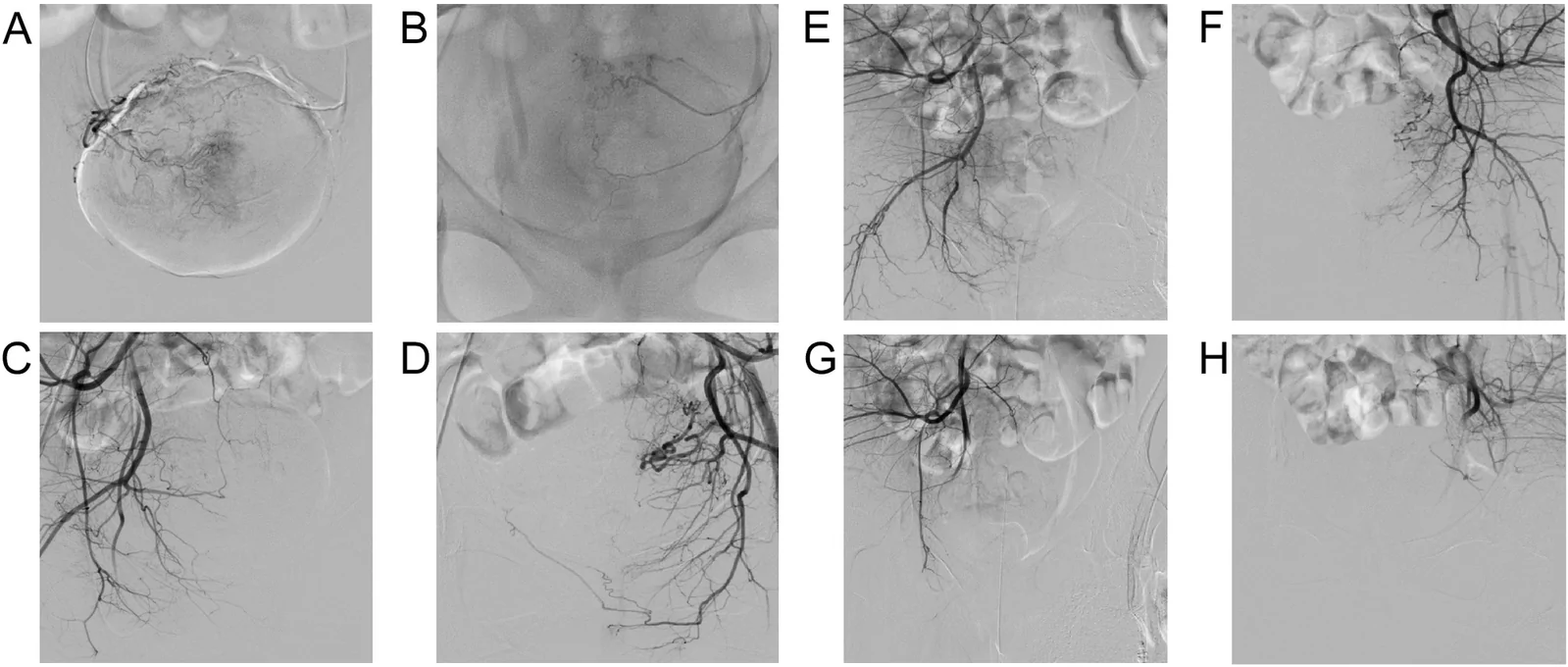
Pelvic arteriography and embolization. First embolization on day 71: right **(A)**, left **(B)**; second on day 74: right **(C)**, left **(D)**; third on day 81: right **(E)**, left **(F)**; and postembolization follow-up: right **(G)**, left **(H)**.

Given the refractory bleeding, immunocompromised state, and multiple viral infections, a combined regimen was initiated on day 84: four weekly intravenous infusions of allogeneic umbilical cord-derived mesenchymal stromal cells (MSCs, 1 ×  10⁶ cells/kg per dose) were administered together with three doses of intravenous cidofovir (5 mg/kg every 7–10 days). The cidofovir dosing interval was extended due to limited drug availability and logistical constraints in mainland China at the time. Baseline serum creatinine before the first cidofovir dose was 43 μmol/L. To minimize nephrotoxicity, rigorous hydration, urinary alkalinization, and probenecid were administered. No acute infusion-related reactions were observed during or after MSC infusions. The MSCs were derived from healthy donor umbilical cord, provided by the Shandong Cord Blood Bank, and manufactured under GMP standards. Supportive care included frequent transfusions, thrombopoietin receptor agonist, G-CSF, fibrinogen, and intravenous immunoglobulin.

A marked clinical response was observed from day 98 onward, with progressive reduction in hematuria and transfusion requirements. By day 124, urinalysis was normalized, and imaging confirmed the resolution of bladder clots (Figure 3). BKV load steadily declined. At 9 months post-transplant, the patient remains asymptomatic with minimal BKV viruria (Figure 4), sustained MRD-negative CR with full donor chimerism, satisfactory hematopoietic recovery (white blood cells, 4.3 ×  10⁹/L; hemoglobin, 91 g/L; platelets, 85 × 10⁹/L), and preserved renal function (serum creatinine, 55 μmol/L). This case suggests that combined MSC and cidofovir therapy may be a viable option for severe, intervention-refractory BKV-HC after HSCT.

**Figure 3:**
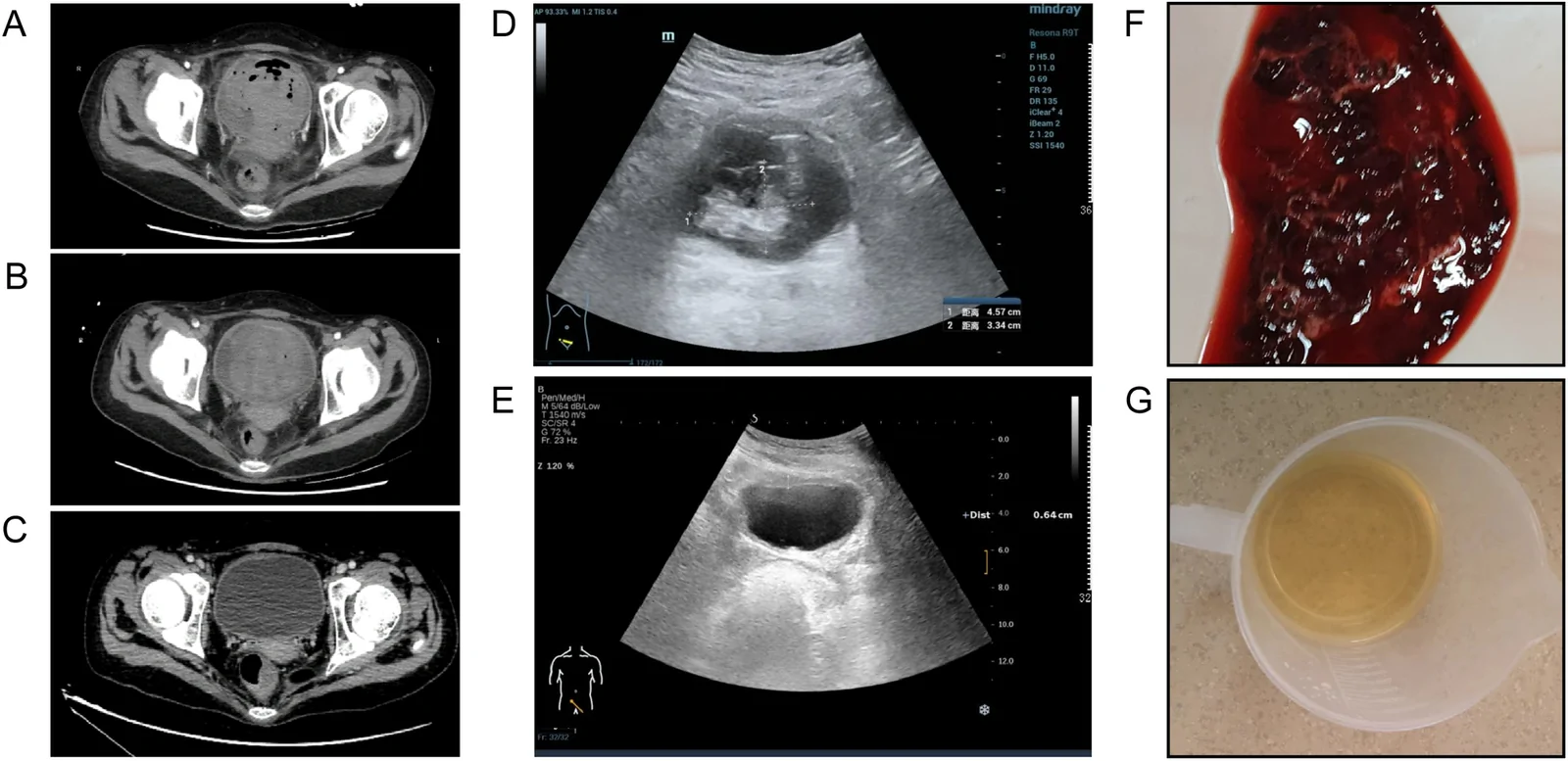
Changes in the bladder on contrast-enhanced abdominal CT and ultrasound before and after cystitis treatment. **(A)** Contrast-enhanced abdominal CT on day 71 showing bladder filled with blood clots and pneumatosis (from irrigation); **(B)** follow-up CT on day 106 showing residual clots with reduced volume; (**C)** follow-up CT on day 124 showing no clots; **(D)** ultrasound on day 72 showing rough, thickened bladder wall with internal clots; **(E)** follow-up ultrasound on day 200 showing no clots and marked improvement in wall thickening; **(F)** urine and clots drained before treatment; and **(G)** clear urine on day 134 without gross hematuria.

**Figure 4:**
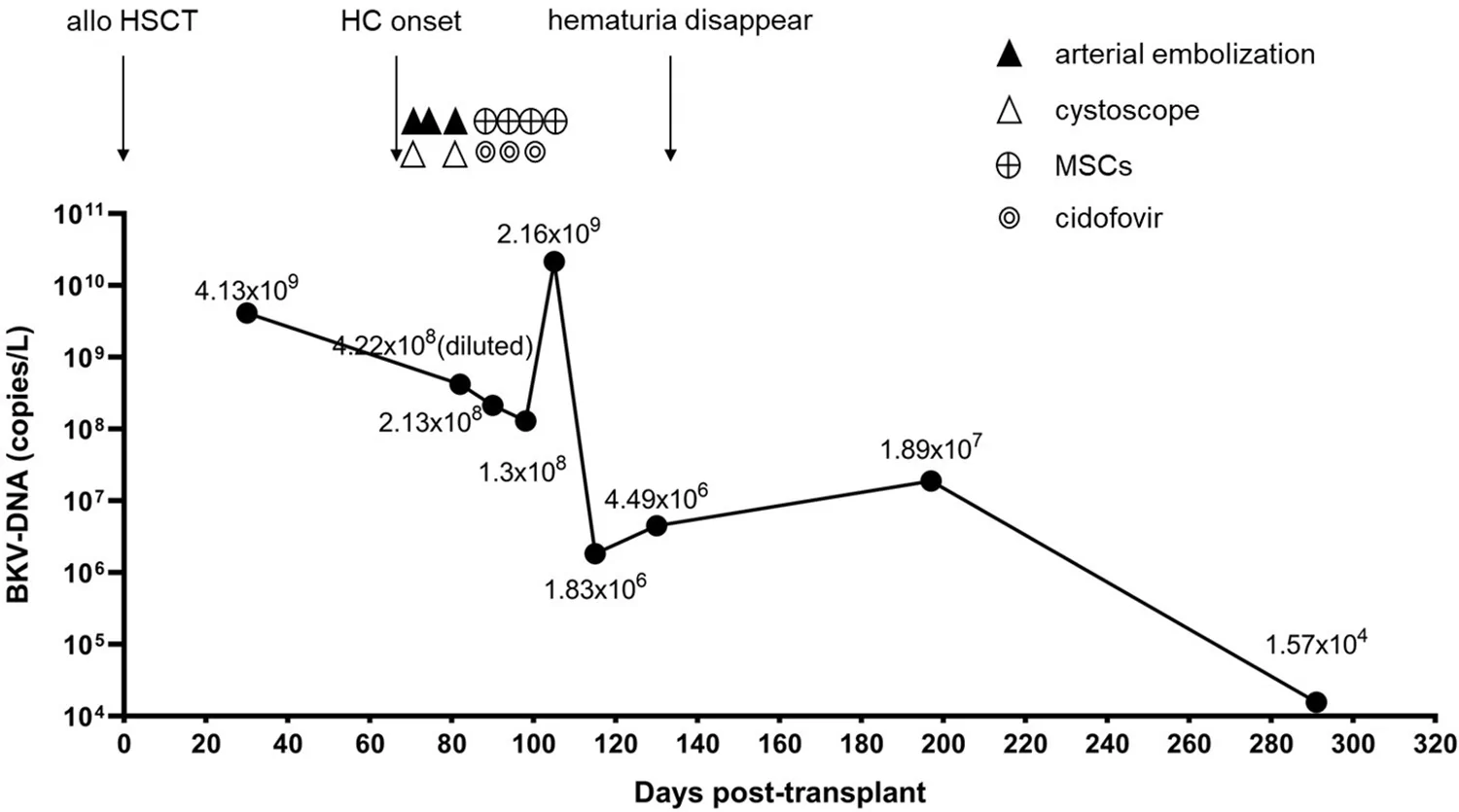
Urinary BK viral load during treatment for BKV-HC.

## Discussion

Severe late-onset HC is predominantly associated with BKV reactivation and contributes substantially to morbidity while also serving as a risk factor for non-relapse mortality (5). Risk factors include intensive conditioning (e.g., with cyclophosphamide), GVHD, haploidentical transplantation, profound immunosuppression, and concurrent viral infections like BKV and CMV (6). Mild HC can be managed with supportive measures, including vigorous hydration, urine alkalinization, diuresis, and analgesia. For moderate-to-severe or virus-associated HC, first-line treatment consists of immunosuppression reduction to enhance viral clearance; second-line options include cystoscopic clot evacuation with electrocautery and interventional arterial embolization to reduce bladder vascular pressure. Antivirals such as cidofovir (intravesical or intravenous) are used to clear the BK virus but are often limited by nephrotoxicity. For refractory cases, emerging therapies such as MSC infusion show promise due to their immunomodulatory and tissue-repair properties (4, 7). Other reported treatments include virus-specific T cells (8, 9), oral leflunomide, ciprofloxacin, estrogen, hyperbaric oxygen therapy (10), and intravesical injections of fibrin glue and platelet-rich plasma. These have been described in small case series with variable efficacy.

The patient in this report presented with multiple high-risk factors: haploidentical HSCT, a cyclophosphamide-containing conditioning regimen, post-transplant CMV infection, and high urine BKV load. The clinical course was further complicated by poor graft function with severe pancytopenia by day 69, which exacerbated bleeding. Cystoscopy revealed widely congested and edematous mucosa with diffuse oozing. Despite maximal conventional therapy—including continuous bladder irrigation, repeated interventional arterial embolization, and cystoscopic electrocautery—hemorrhage remained uncontrolled. We acknowledge the potential risk of ischemia and necrosis associated with repeated bladder arterial embolization. However, all embolization procedures in this case were superselective, using appropriately sized particles (gelatin sponge, blank microspheres, and PVA particles) and targeting only the bleeding vessels rather than the entire bladder vascular supply. With this approach, the risk of extensive bladder wall ischemia was considered limited. Hyperbaric oxygen therapy was considered but not used, given the patient's severe thrombocytopenia and active bleeding. After multidisciplinary discussion, we opted for a combination regimen of MSCs and cidofovir rather than more aggressive options such as urinary diversion or cystectomy, as this approach was considered more suitable for the patient's complex condition of HC complicated by severe thrombocytopenia and concurrent viral infections, particularly BK virus. Intravenous immunoglobulin (20 g weekly) was also added for immunomodulation.

MSCs support hematopoiesis and exert immunomodulatory effects. They promote hematopoietic stem cell engraftment and recovery through cytokine secretion (11, 12) and modulate immune responses via soluble factors and cell contact, inhibiting effector lymphocytes while promoting regulatory T cells (13). In 2007, Ringdén et al. reported the use of bone marrow-derived MSCs in seven patients with post-transplant hemorrhagic cystitis. Following MSC infusion (0.7–1.6 × 10⁶/kg, 2–5 doses), gross hematuria resolved in five patients (grade 2–3, using the NCI terminology for adverse events) at a median of 3 days, with microscopic hematuria clearing by a median of 23 days; the remaining two patients developed grade 4–5 hemorrhagic cystitis and eventually died (14). A 2015 trial of placental-derived MSCs in seven patients with severe late-onset cystitis demonstrated resolution of gross hematuria in three cases and no obvious response in two cases, whereas the remaining two were difficult to evaluate. No infusion toxicity or increased infection risk was observed. Nevertheless, three patients ultimately died due to GVHD or severe infection (15). A retrospective study of 13 children with grade III–IV HC after cord-blood transplantation reported universal improvement after weekly MSC infusions (1 × 10⁶/kg), including eight cures. The median time to resolution was 10 days (range, 1–25) for gross hematuria and 13 days (range, 1–27) for microscopic hematuria following MSC infusion. Although BKV replication was unaffected, efficacy was attributed to MSC-mediated tissue repair and immunomodulation. However, four patients ultimately died from GVHD, relapse, or infection (7). Preclinical studies further suggest that exosomes from immortalized MSCs can also promote bladder mucosal recovery (16, 17). It is evident that reports on the use of MSCs for HC remain very limited. Moreover, many patients with severe HC often have concurrent transplant-related complications, such as GVHD or infections, which makes it difficult to fully evaluate the therapeutic effect of MSCs on cystitis due to interference from these factors.

In this case, umbilical cord-derived MSCs (1 × 10⁶/kg/dose) were administered weekly for four doses starting on day 84 post-transplant. Clinical improvement was evident approximately on day 98, with reduced pain, decreased clot burden, and declining transfusion needs. Gross hematuria resolved completely on day 124 (40 days after combination therapy initiation), and microscopic hematuria cleared by day 127. Hematopoietic recovery was also notable, with transfusion independence achieved by day 131 post-transplant (Figure 5). At 9 months, blood counts were stable, with sustained complete remission and full donor chimerism. However, this recovery was likely multifactorial—potentially attributable to a combination of immunosuppression tapering, administration of hematopoietic growth factors, resolution of viral infection, and MSC infusion—and the individual contribution of each factor cannot be determined in this single case.

**Figure 5:**
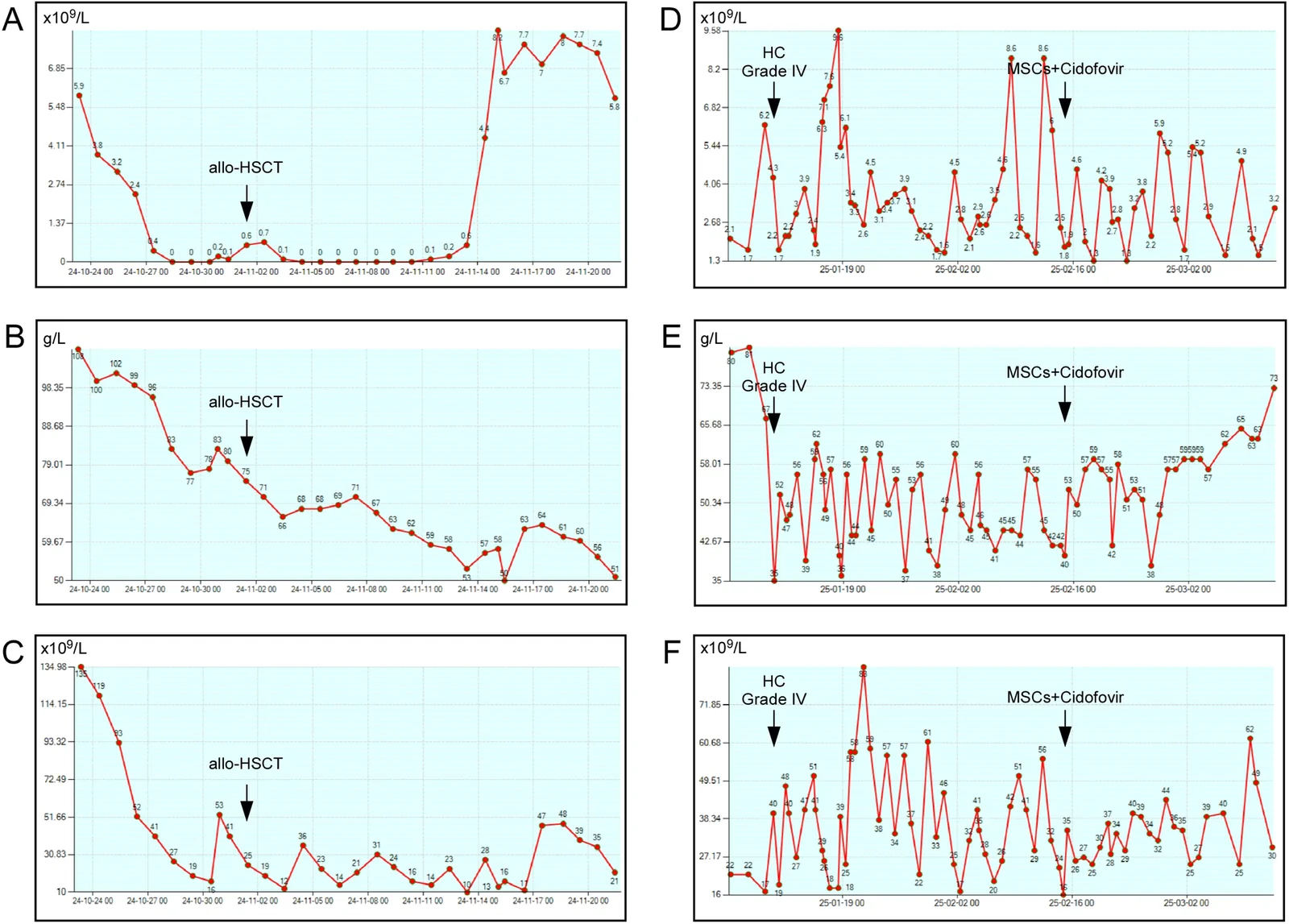
Trends in blood cell counts before and after transplantation and during cystitis treatment. **(A–C)** Trends in white blood cell count **(A)**, hemoglobin **(B)**, and platelet count **(C)** before and after transplantation. **(D–F)** Trends during treatment for grade IV delayed hemorrhagic cystitis: white blood cell count **(D)**, hemoglobin **(E)**, and platelet count **(F)**.

BKV is ubiquitous, with primary infection usually asymptomatic. However, immunocompromised patients, especially after allo-HSCT, are at significant risk of reactivation and may develop BKV-HC. In monitored HSCT recipients, viral DNA in urine increases from <10% pretransplant to 50%–100% post-transplant, with the incidence of BKV-HC around 16% (range 7%–54%) in adults (18). Several antivirals have been studied, including fluoroquinolones (19), leflunomide (20), cidofovir (intravenous or intravesical) (21, 22), and virus-specific T-cell therapy. Cidofovir, a broad-spectrum nucleotide analog, is the most widely investigated. A systematic review of nine studies (184 allo-HSCT patients with HC) reported complete response in 68% of 172 patients receiving intravenous cidofovir and 88% of 17 treated with intravesical instillation, although study heterogeneity limits comparability (2). Nephrotoxicity is a known risk of intravenous cidofovir, with incidences ranging from 17% to 36% across different studies. Intravesical administration, however, appears to only modestly reduce this risk (23). For prophylaxis, a study by Lv et al. found that preemptive cidofovir significantly lowered BKV load and HC incidence in allo-HSCT patients compared to conventional management (24).

In this case, by day 30 post-transplant, high-level BKV was detected in the urine, without significant symptoms. On day 69, the patient progressed to grade IV HC, with BKV-DNA in bladder irrigation fluid measuring 4.22 × 10⁸ copies/L. Starting on day 84, cidofovir was initiated alongside umbilical cord-blood-derived MSCs to reduce inflammation and support recovery. By day 98, urinary pain decreased, and transfusion needs dropped. Urinary BKV load decreased significantly by day 116, and gross hematuria resolved by day 124. At 9 months post-transplant, BKV load was very low. The patient had previously developed acute kidney injury from liposomal amphotericin B, which resolved with supportive care. During cidofovir therapy, aggressive hydration, urinary alkalinization, and probenecid were used to mitigate nephrotoxicity. No renal impairment occurred.

To date, no reports have described the combined use of MSCs and cidofovir for BKV-associated hemorrhagic cystitis. One retrospective study reported that antiviral agents (including cidofovir, ganciclovir, and foscarnet) failed to control refractory late-onset HC, and MSCs were subsequently used as salvage therapy, showing a favorable overall response (CR rate 85%); however, in patients with grade IV HC, the CR rate was only 50% (25). This suggests that salvage MSC therapy after antiviral failure remains suboptimal for severe HC. In the present case, we applied MSCs and cidofovir concurrently as a planned combination strategy, rather than as a sequential rescue measure after antiviral failure. This combination was based on a dual-target rationale: cidofovir for viral control and MSCs for bladder mucosal repair via immunomodulation and paracrine effects, as well as to facilitate hematopoietic recovery. This approach represents an exploratory effort to optimize combination therapy for patients with complex, refractory clinical conditions.

A summary of the clinical course is presented in Figure 6.

**Figure 6:**
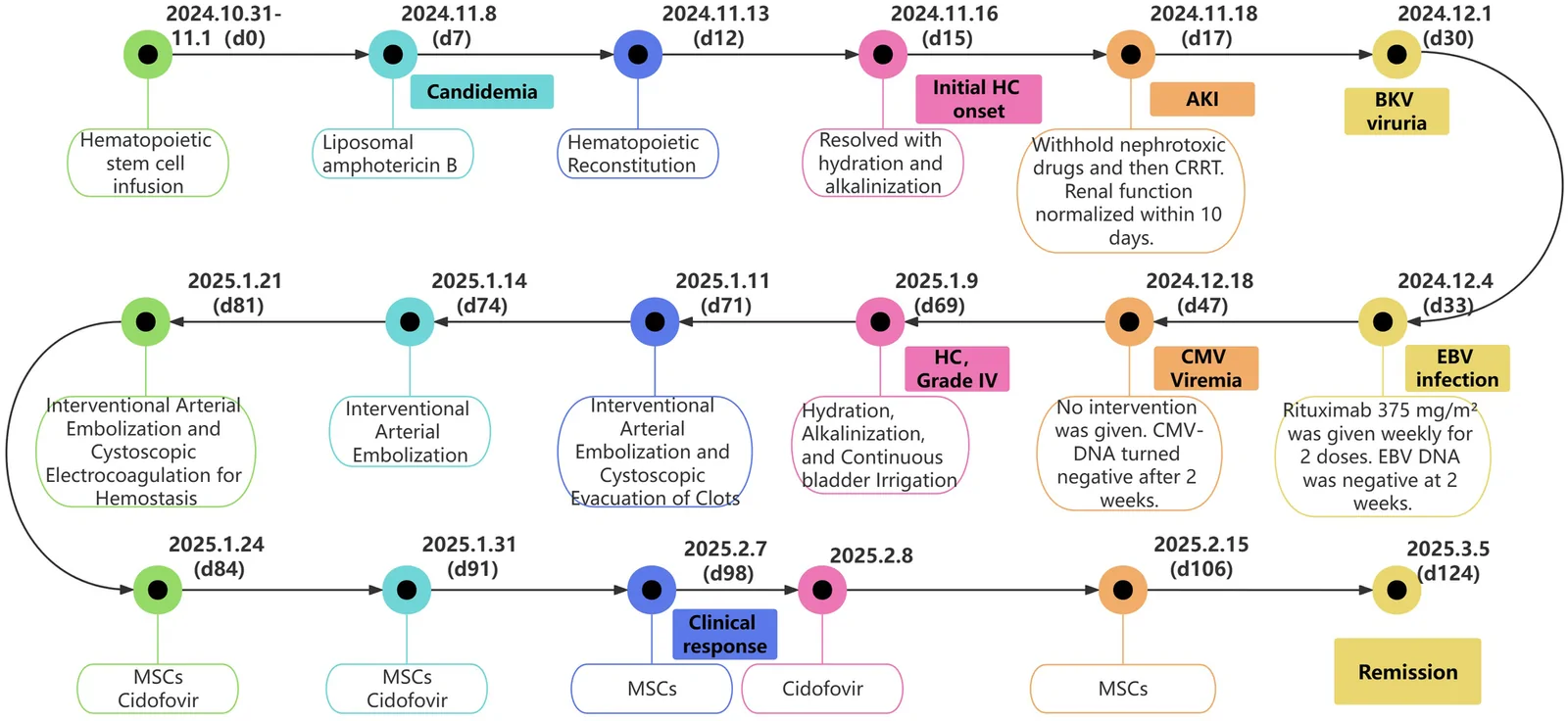
Post-transplantation infection and HC treatment course.

### Limitations

Several limitations of this case report should be acknowledged. First, as a single case report, the findings cannot be generalized to all patients with BKV-associated HC following allogeneic HSCT because treatment response may vary with individual factors such as immune reconstitution status, viral load, and the degree of graft dysfunction. Second, the concurrent use of MSCs and cidofovir makes it impossible to determine the individual contribution of each therapy to the observed clinical improvement. Third, urine PCR for CMV and adenovirus was not performed, which limits our ability to definitively rule out other viral etiologies of HC; however, the diagnosis of BKV-HC was strongly supported by positive urine BKV DNA and a clinical presentation consistent with typical post-transplant BKV-associated HC. Fourth, although the temporal association between initiation of combination therapy and clinical improvement was suggestive, we cannot entirely rule out spontaneous resolution, particularly given that immunosuppression tapering may have contributed to graft recovery, viral clearance, and symptom improvement. In addition, rituximab administered on days 34 and 41 may have contributed to immunomodulation and should be considered a potential confounder, although its direct role in BKV-HC after allogeneic HSCT remains unclear. Given these cointerventions, the observed clinical benefits cannot be attributed solely to the MSC-plus-cidofovir combination. Fifth, the follow-up duration is relatively limited (9 months), which restricts assessment of long-term outcomes, including late relapse of hemorrhagic cystitis, delayed viral reactivation, and potential late-onset adverse effects of MSCs such as ectopic tissue formation or malignant transformation. Finally, while our patient demonstrated favorable hematopoietic recovery and preserved renal function, the optimal dosing, timing, and sequence of combined MSC and cidofovir therapy remain to be defined. Larger prospective studies are needed to validate these preliminary observations.

## Conclusion

This case demonstrates that combined MSCs and cidofovir therapy can be an effective salvage strategy for severe, refractory BKV-HC after allo-HSCT. The approach likely targets both the viral trigger and the associated inflammatory tissue injury, as well as poor graft function. While promising, this strategy requires further prospective evaluation to optimize its timing, dosing, and application.

## Data Availability

The original contributions presented in the study are included in the article/Supplementary Material, further inquiries can be directed to the corresponding author.
